# Secretome of SHED Sheets Facilitates Mandibular Bone Defect Repair by Shifting Macrophages Inflammatory State

**DOI:** 10.1016/j.identj.2026.109793

**Published:** 2026-08-18

**Authors:** Xi Xiang, Wenbin Wu, Wenyan Huang, Danyuan Xie, Jianhong Zeng, Jianwen Li, Yuhang Huang, Keyu Lv, Ying Ruan, Luwei Wang, Changhua Hao, Janak L Pathak, Sujuan Zeng, Bo Peng

**Affiliations:** a Department of Pediatric dentistry, School and Hospital of Stomatology, Guangdong Engineering Research Center of Oral Restoration and Reconstruction, and Guangzhou Key Laboratory of Basic and Applied Research of Oral Regenerative Medicine, Guangzhou Medical University, Guangzhou, China; b Department of Stomatology, Key Laboratory of Biological Targeting Diagnosis, Therapy and Rehabilitation of Guangdong Higher Education Institutes, The Fifth Affiliated Hospital, Guangzhou Medical University, Guangzhou, China

**Keywords:** Stromal cells from human exfoliated deciduous teeth, Bone regeneration, Macrophage polarization, Immunomodulation, Secretome

## Abstract

**Objective:**

The inflammatory response, especially the inflammatory status of macrophages, is crucial for bone graft integration and regeneration. Cell sheets from various stromal cells exhibit immunomodulatory and pro-osteogenic traits. However, the therapeutic potential of the secretome from human exfoliated deciduous teeth stromal cell (SHED) sheets remains unclear. This study aimed to explore whether SHED sheet-derived secretome (SS-E) aids bone defect repair via macrophage immunomodulation and to clarify the underlying mechanisms.

**Methods:**

SS-E was collected from osteogenically induced SHED sheet supernatants. Its immunomodulatory effects on lipopolysaccharide (LPS)-stimulated inflammatory macrophages were studied using mRNA sequencing and analysis of polarization marker expression. Paracrine osteogenic potential was evaluated by culturing bone marrow mesenchymal stromal cells (BMSC) in the conditioned medium from SS-E-primed macrophages (SS-E-CM). Osteogenic differentiation was quantified through Western blotting, RT-qPCR, alkaline phosphatase (ALP) staining, and Alizarin Red S staining. In vivo, a rat mandibular bone defect model was treated with gelatin methacryloyl (GelMA) hydrogel or GelMA loaded with SS-E (GelMA + SS-E). Bone regeneration was assessed using micro-CT, H&E staining, and immunohistochemistry.

**Results:**

SS-E treatment significantly decreased the M1 pro-inflammatory marker iNOS in LPS-stimulated macrophages and increased M2 anti-inflammatory markers (Arg-1, IL*-*10, CD206). SS-E-CM enhanced BMSC proliferation and upregulated osteogenic factors (RUNX2, ALP, BMP-2), with increased ALP activity and matrix mineralization. In vivo, GelMA + SS-E reduced local inflammation and enhanced new bone formation compared to GelMA alone.

**Conclusion:**

The SS-E promotes osteogenesis indirectly by redirecting macrophage polarization from a pro-inflammatory to a pro-reparative phenotype. This cell-free, immunomodulatory strategy represents a promising therapeutic approach for complex craniofacial bone reconstruction.

**Translational impact statement:**

This study presents SS-E, a cell-free secretome derived from stromal cells of human exfoliated deciduous teeth, which safely modulates macrophage-driven inflammation to promote bone regeneration. By combining SS-E with a clinically compatible hydrogel, this immunomodulatory strategy offers a readily translatable approach for enhancing craniofacial bone defect repair without the risks associated with cell-based therapies.

## Graphical abstract

**Figure:**
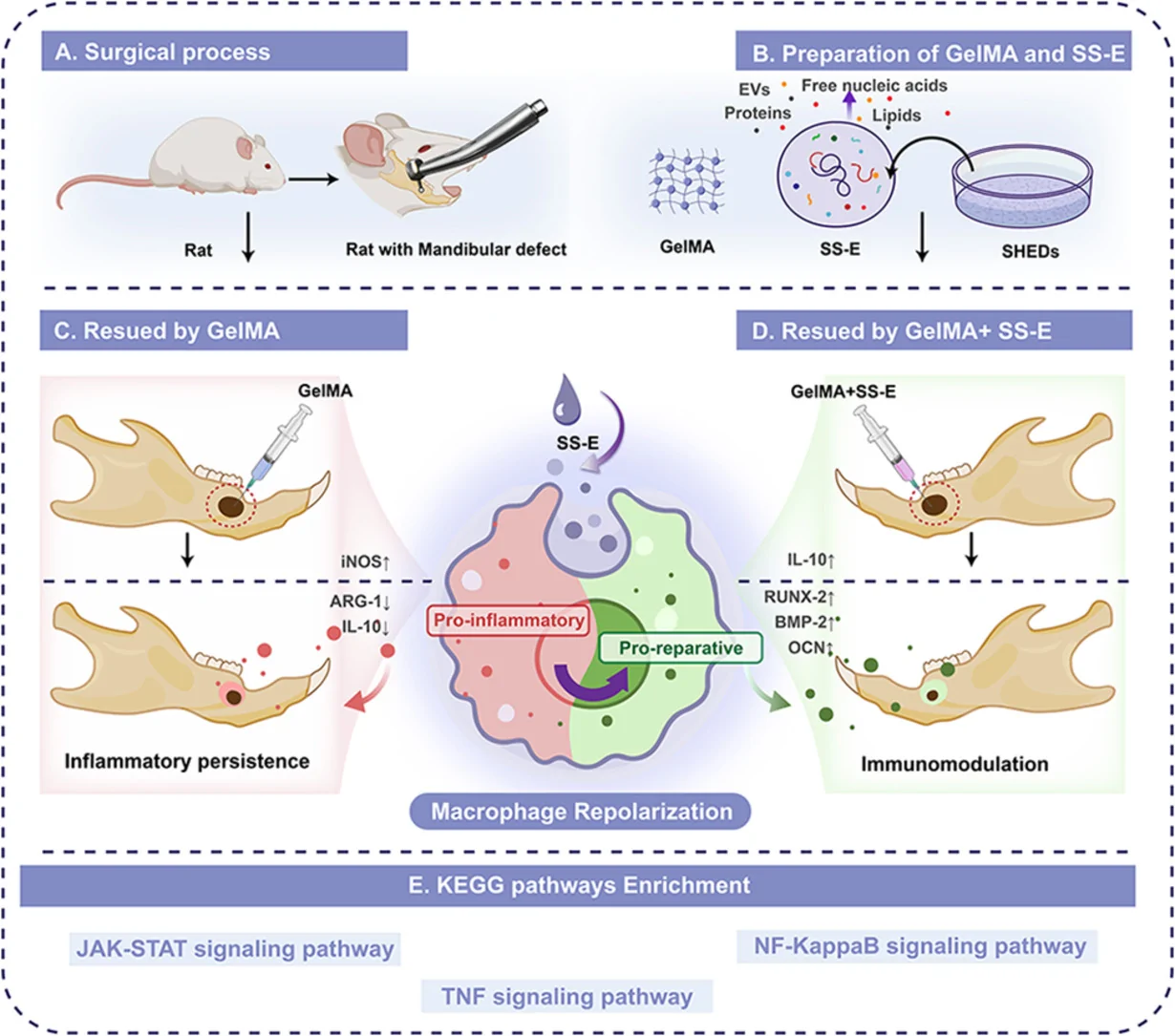

## Introduction

The reconstruction of mandibular defects resulting from trauma, oncologic resection, or congenital anomalies remains a significant clinical challenge.^1^ While hydrogel-based scaffolds such as gelatin methacryloyl (GelMA) have advanced the field of bone tissue engineering, their clinical utility is constrained by the host immune response, which recognizes these materials as foreign and can provoke adverse inflammatory reactions that compromise regeneration.^2^

Mesenchymal stromal cell-derived secretomes (MSC-E), the collection of bioactive factors secreted by mesenchymal stromal cells (MSC), have emerged as a promising cell-free therapeutic alternative. By circumventing the risks associated with direct cell transplantation, including immune rejection and tumorigenicity, MSC-E offers an enhanced safety profile while delivering a concentrated array of growth factors, cytokines, and extracellular vesicles (EV) that promote tissue repair.^3^, ^4^, ^5^ Notably, when MSC are cultured at high density without enzymatic dissociation, they form cohesive cell sheets that secrete an extracellular matrix-enriched supernatant with a greater abundance of trophic factors and higher expression of stemness-associated genes than in conventional two-dimensional cultures.^6^, ^7^, ^8^

Among MSC populations, stromal cells from human exfoliated deciduous teeth (SHED) are particularly attractive for craniofacial applications due to their facile, non-invasive isolation, robust proliferative capacity, potent osteogenic potential, and superior immunomodulatory properties relative to adult MSC.^9^^,^^10^ Furthermore, osteogenic induction has been shown to enhance both sheet formation and the secretory profile of SHED sheets (SS).^11^^,^^12^ Mounting evidence indicates that dental pulp-derived stromal cells exert bone-regenerative effects by modulating macrophage inflammatory behavior in pathological microenvironments. A recent study further confirmed that macrophage-dental stromal cell crosstalk is a key regulatory axis in inflammatory bone lesions, and this interaction can be modulated by bioactive agents, including ginsenoside RB1.^13^ However, the therapeutic potential of the secretome derived from osteogenically induced SHED sheets (SS-E) in modulating macrophage-mediated inflammation and promoting bone repair remains unexplored.

Given that macrophage-conditioned medium effectively recapitulates the inflammatory microenvironment *in vitro*,^14^ it is both relevant and timely to investigate whether SS-E can shift the macrophage phenotype from a pro-inflammatory to a pro-reparative state, thereby creating a favorable osteoimmune niche. Accordingly, this study was designed to: (i) establish a robust method for generating SS-E from osteogenically induced SHED sheets; (ii) characterize the immunomodulatory effects of SS-E on macrophage polarization; and (iii) evaluate the osteogenic efficacy of SS-E delivered via a GelMA hydrogel scaffold in a rat model of mandibular bone defect repair.

## Materials and methods

### Cell culture and identification

Human SHED were isolated from the deciduous teeth (incisors and canines) of three healthy donors (aged 6-8 years) with informed consent. Cells from all donors were pooled, used fresh or cryopreserved, and passages 3-6 were used. Rat BMSC were isolated from the femurs and tibias of male SD rats (4 weeks old). RAW264.7 macrophages were purchased from Procell (CL-0190, China). SHED and BMSC were cultured in DMEM with 10% FBS and 1% penicillin–streptomycin; RAW264.7 cells were maintained in DMEM with 10% heat-inactivated FBS and 1% penicillin–streptomycin, all at 37 °C with 5% CO₂. To induce LPS-stimulated inflammatory macrophages, RAW264.7 cells were treated with 100 ng/mL LPS for 24 h to establish an *in vitro* inflammatory cell model mimicking the local inflammatory microenvironment of traumatic bone defects.

### Flow cytometry analysis

SHED were analyzed for mesenchymal (CD73, CD90, CD105) and hematopoietic (CD19, CD34, CD45) markers by flow cytometry (BioLegend, USA). BMSC were analyzed for CD29, CD44, CD90 (positive), and CD31, CD34, CD45 (negative) (BioLegend, USA). For M2 macrophage polarization, RAW264.7 cells were stained with anti-CD206-PE (BioLegend, USA) after live/dead staining (eBioscience, USA) and Fc blocking (BD Biosciences, USA). Data were acquired on a BD FACSCanto II (BD Biosciences, USA) and analyzed with FlowJo.

### Preparation of SHED sheet-derived secretome (SS-E)

To generate SS, SHED were cultured in osteogenic mineralization medium (OM) consisting of DMEM supplemented with 10% FBS, 50 μg/mL ascorbic acid, 10 mM β-glycerophosphate, and 10 nM dexamethasone for 10 days until dense sheets formed. After sheet formation, the medium was replaced with serum-free mesenchymal stromal cell basal medium (iCell, China), and conditioned medium was collected every 24 h for three consecutive days, with fresh medium added after each collection. The collected media were pooled, centrifuged (1000 rpm, 5 min), filtered (0.22 μm), and concentrated using 3 kDa cutoff centrifugal filters (Millipore, Germany) at 5000 × g for 1 h at 4 °C. The concentrated secretome was quantified by BCA assay and stored at −80 °C until use. All SS-E samples were prepared according to a standardized protocol to ensure batch consistency.

### Preparation of macrophage-conditioned media (CM and SS-E-CM)

To generate conditioned media for BMSC cultures, LPS-stimulated inflammatory macrophages were cultured for 24 h either in the absence or presence of the optimal concentration of SS-E. After 24 h, the culture medium was removed, cells were washed gently with Phosphate-buffered saline (PBS), and incubated in serum-free DMEM for an additional 6 h. The supernatants were collected from multiple culture wells, pooled to minimize batch-to-batch variation, and centrifuged at 1000 rpm for 5 min to remove cellular debris. The clarified supernatants were then filtered through a 0.22 μm filter and immediately mixed 1:1 (v/v) with OM. This process yielded two distinct conditioned media: CM (derived from LPS-stimulated inflammatory macrophages without SS-E treatment) and SS-E-CM (derived from LPS-stimulated inflammatory macrophages treated with SS-E). For BMSC experiments, pure OM served as the control group (Con). All conditioned media were prepared fresh for each experiment.

### Cell viability assay

Cells were seeded at a density of 3000 cells per well in 96-well plates and cultured under appropriate conditions. After treatment, 10% (v/v) CCK-8 solution (CCK-8; Dojindo, Japan) was added to each well and incubated in the dark for 2 hours. Absorbance was measured at 450 nm using a SPECTROstar Nano microplate reader. For RAW264.7 cells, viability was assessed at different SS-E concentrations; for BMSC, viability was evaluated in response to CM and SS-E-CM.

### Immunofluorescence staining

After LPS-stimulated inflammatory macrophages and SS-E treatment, RAW264.7 cells on coverslips were fixed, blocked, and stained for CD206 (Proteintech, China) overnight at 4°C, followed by fluorescent secondary antibody (1 h) and DAPI. Images were acquired on a Leica confocal microscope (excitations: 405/552 nm).

### RT-qPCR

Total RNA from RAW264.7 cells or BMSC was extracted using the SteadyPure Universal RNA Extraction Kit (Accurate Biotechnology, China) and reverse-transcribed into cDNA with the PrimeScript® RT Reagent Kit (Takara, Japan). Quantitative real-time PCR (RT-qPCR) was then performed on a Bio-Rad system using SYBR Premix Ex Taq™ (Tsingke, China), with GAPDH as the reference gene. The primer sequences and amplification efficiency are listed in Table 1. All assays included 3 biological replicates and 3 technical replicates to guarantee data robustness, and a full MIQE compliance checklist covering all required reporting items is provided in Supplementary Table 1.

**Table 1:** Primers used for RT-qPCR analysis.

Gene	Acc. No.	Primer sequence (5’-3’)	Product length (bp)	Amplification efficiency (%)
*Mu GAPDH*	NM 001289726.1	Forward: AAGAAGGTGGTGAAGCAGG	111	98.5%
		Reverse: GAAGGTGGAAGAGTGGGAGT		
*Mu Inos*	NM 001313922.1	Forward: ACTCAGCCAAGCCCTCA	105	102.3%
		Reverse: CTCTGCCTATCCGTCTCGT		
*Mu Arg1*	NM 007482.3	Forward: GATTATCGGAGCGCCTTTCT	147	95.7%
		Reverse: CCACACTGACTCTTCCATTCTT		
*Mu Il10*	NM 010548.2	Forward: ACTGGCATGAGGATCAGCAG	119	101.2%
		Reverse: CTCCTTGATTTCTGGGCCAT		
*Ra Runx2*	NM 001278484.3	Forward: GCCGGGAATGATGAGAACTA	182	97.8%
		Reverse: GAGGCAGAAGTCAGAGGTGG		
*Ra Alpl*	NM 013059.3	Forward: ATCGGACCCTGCCTTACCAA	81	99.1%
		Reverse: GGTCTCTTGGGCTTGCTGTC		
*Ra Bmp2*	NM 017178.2	Forward: AGTAGTTTCCAGCACCGAATTA	99	103.4%
		Reverse: CACTAACCTGGTGTCCAATAGT		
*Ra GAPDH*	NM 017008.4	Forward: ACGGGAAACCCATCACCATC	206	96.3
		Reverse: CTCGTGGTTCACACCCATCA		

Note: Amplification efficiency for each primer pair via standard curve dilution; all values fell within 90% to 110%, complying with the MIQE standard range. *GAPDH* served as the reference gene: Cq analysis yielded CV < 2% under both LPS-induced inflammatory and osteogenic induction conditions, verifying its stable expression.

### Western blot analysis

Protein samples (10 μg) extracted from RAW264.7 cells or BMSC were separated by SDS-PAGE using 10% separating gel and transferred to PVDF membranes. After blocking with QuickBlock™ Buffer (Beyotime, China) for 30 min, the membranes were incubated overnight at 4 °C with primary antibodies against GAPDH, iNOS, Arg-1, ALP, BMP-2, and RUNX2 (Abcam, UK). Following incubation with corresponding secondary antibodies (Thermo Fisher, USA) for 1 h at room temperature, protein bands were visualized using an ECL kit and quantified with ImageJ. Protein band intensity was normalized to GAPDH as the loading reference.

### Analysis of osteogenic mineralization

BMSC were treated with SS-E-CM, CM, or OM at 70% to 80% confluence. After 7 days, ALP activity was assessed by fixing cells in 4% paraformaldehyde, followed by staining with BCIP/NBT solution (Beyotime, China) for 20 minutes at room temperature in the dark. The staining was imaged and quantified in ImageJ. Mineralization was assessed on Day 14 by ARS staining (Solarbio, China). After fixation and staining, nodules were imaged. For quantification, nodules were dissolved in 10% cetylpyridinium chloride (CPC), and eluent absorbance was measured at 562 nm.

### mRNA sequencing

Transcriptome sequencing was performed by Shanghai Applied Protein Technology. After quality control, high-quality reads were aligned to the reference genome. Genes with |Log₂(fold change)| ≥ 1 and adjusted *P*-value (padj) < 0.05 were defined as differentially expressed genes for subsequent enrichment analysis. Key results were validated by RT‑qPCR.

### GelMA hydrogel preparation

Gelatin methacryloyl (GelMA; EFL, China) served as the SS-E carrier. A 10% (w/v) GelMA solution was sterilized by autoclaving, maintained at 37 °C, and mixed with 1.6 mg/mL SS-E (or PBS for controls) before being molded into 3 × 3 × 3 mm constructs. Cross-linking was induced under UV light for 2 minutes.

### Animal experiment design

All animal experiments were conducted in strict accordance with the ARRIVE2 guidelines (Supplementary table 2) and complied with the Guide for the Care and Use of Laboratory Animals.

Fourteen male Sprague-Dawley rats (7 per group, 8 weeks old) were obtained from Southern Medical University. For surgical anesthesia, a mixture of tiletamine-zolazepam (Zoletil) and xylazine (1:1, diluted 10-fold in sterile saline) was administered intraperitoneally (0.01 mL/g). A critical-sized mandibular defect (3 × 3 × 3 mm) was created in the unilateral molar region of each rat using a slow-speed drill under saline irrigation. The defects were randomly allocated into two groups and filled with either GelMA+SS-E (experimental group, containing SS-E) or GelMA (control group, containing equal-volume PBS), then covered with a Bio-Gide membrane (Geistlich, Switzerland). The surgical site was closed in layers with absorbable sutures (4-0 Vicryl).

At 6 weeks post-operation, rats were euthanized by CO₂ inhalation. Mandibles were harvested, fixed, and evaluated by micro-CT, followed by histological analyses including H&E staining, Masson's trichrome staining, and immunohistochemistry for iNOS, Arg-1, and OCN.

### Statistical analysis

All experiments were performed in triplicate, and data are presented as mean ± SD. The normality of all datasets was verified using the Shapiro-Wilk test prior to statistical comparisons. Statistical analyses were conducted using GraphPad Prism 9.5.1 (GraphPad Software, USA). Comparisons between two groups were assessed by an unpaired Student's t-test, and one-way ANOVA with Tukey’s post hoc test was adopted for comparisons among three or more groups. A value of *P < 0.05* was considered statistically significant.

## Results

### Identification of SHEDs and BMSCs

**Fig. 1:**
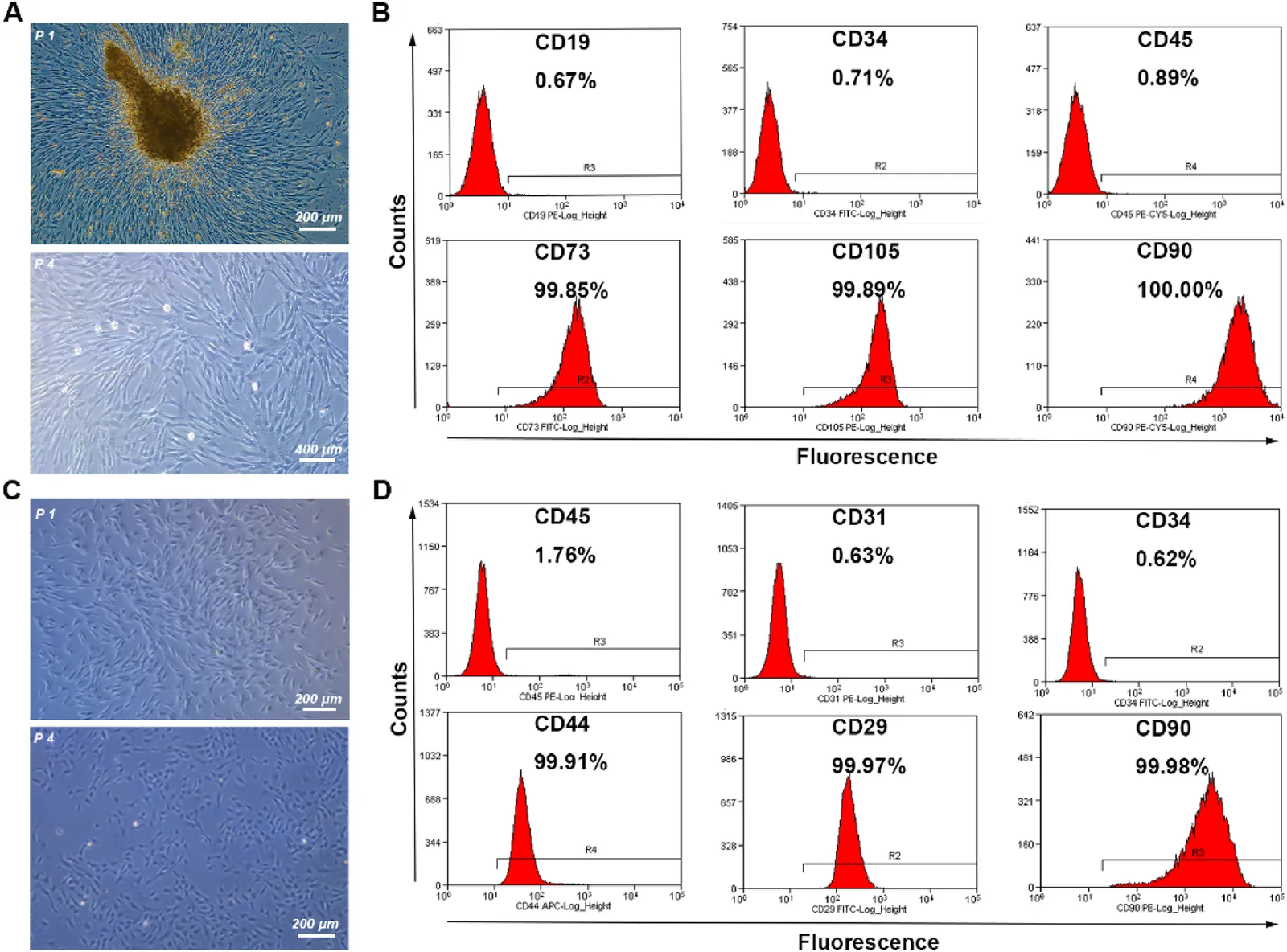
Identification of SHED and BMSC. (A) Morphology of SHED under a microscope. (B) Flow cytometry analysis of surface markers expressed by SHED. (C) Morphology of BMSC under a microscope. (D) Flow cytometry analysis of surface markers expressed by BMSC.

Both SHED and BMSC displayed a typical spindle-shaped morphology (Figure 1A, C) and expressed standard MSC surface markers (CD44/CD73/CD90/CD105+) while lacking hematopoietic markers (CD31/CD34/CD45−) (Figure 1B, D), consistent with international MSC standards.

### SS-E mitigates LPS-induced macrophage inflammation

**Fig. 2:**
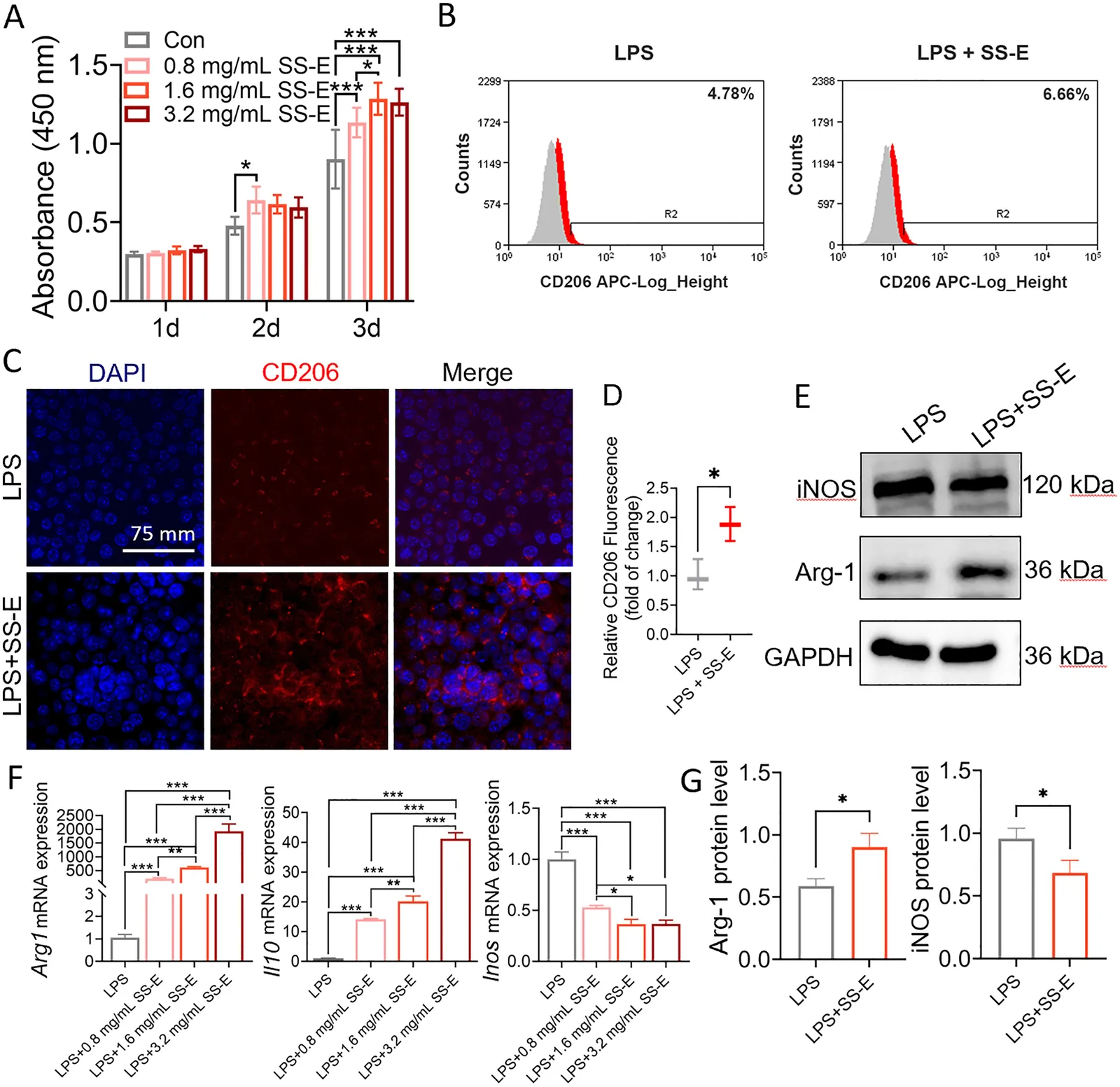
Effects of SS-E on RAW264.7 macrophage viability and immunomodulation. (A) Cell viability was analyzed by CCK-8 assay (n = 3). (B) Representative images of flow cytometry detection of CD206 positivity, a marker of M2 polarization in macrophages. (C) Immunofluorescence analysis of M2 macrophage marker CD206. (D) Quantification of relative CD206 fluorescence intensity, expressed as fold change compared to the LPS group. Representative western blot images (E) and quantification (G) of inflammation regulatory markers (n = 3). (F) RT-qPCR analysis of inflammation regulatory markers (n = 3). Significant differences between the groups, *P<0.05, **P<0.01, ***P<0.001.

CCK-8 assay results (Figure 2A) showed that SS-E (0.8, 1.6, and 3.2 mg/mL) did not suppress RAW264.7 cell proliferation over 1-3 days. Notably, SS-E at 1.6 mg/mL at 48 h and all three concentrations at 72 h significantly enhanced proliferation compared to controls. RT-qPCR (Figure 2F) revealed that, compared with the LPS group, all tested SS-E concentrations significantly downregulated *Inos* and upregulated *Arg1* and *Il10*. Based on the combined assessment of cell viability and anti-inflammatory efficacy, 1.6 mg/mL was selected as the optimal concentration for subsequent experiments, as it exhibited the most favorable balance between promoting proliferation and suppressing inflammation, with no additional benefit at 3.2 mg/mL. Flow cytometry (Figure 2B) and immunofluorescence (Figure 2C, D) consistently showed elevated CD206 expression in the LPS+SS-E group. Western blot (Figure 2E, G) further confirmed that SS-E downregulated iNOS and upregulated Arg-1 at the protein level. Collectively, these results demonstrate that 1.6 mg/mL SS-E effectively mitigates LPS-induced macrophage inflammation. Three independent batches of SS-E were prepared using identical protocols, and comparable biological activities were observed across batches.

### SS-E modulates the inflammatory gene expression profile of LPS-stimulated macrophages

**Fig. 3:**
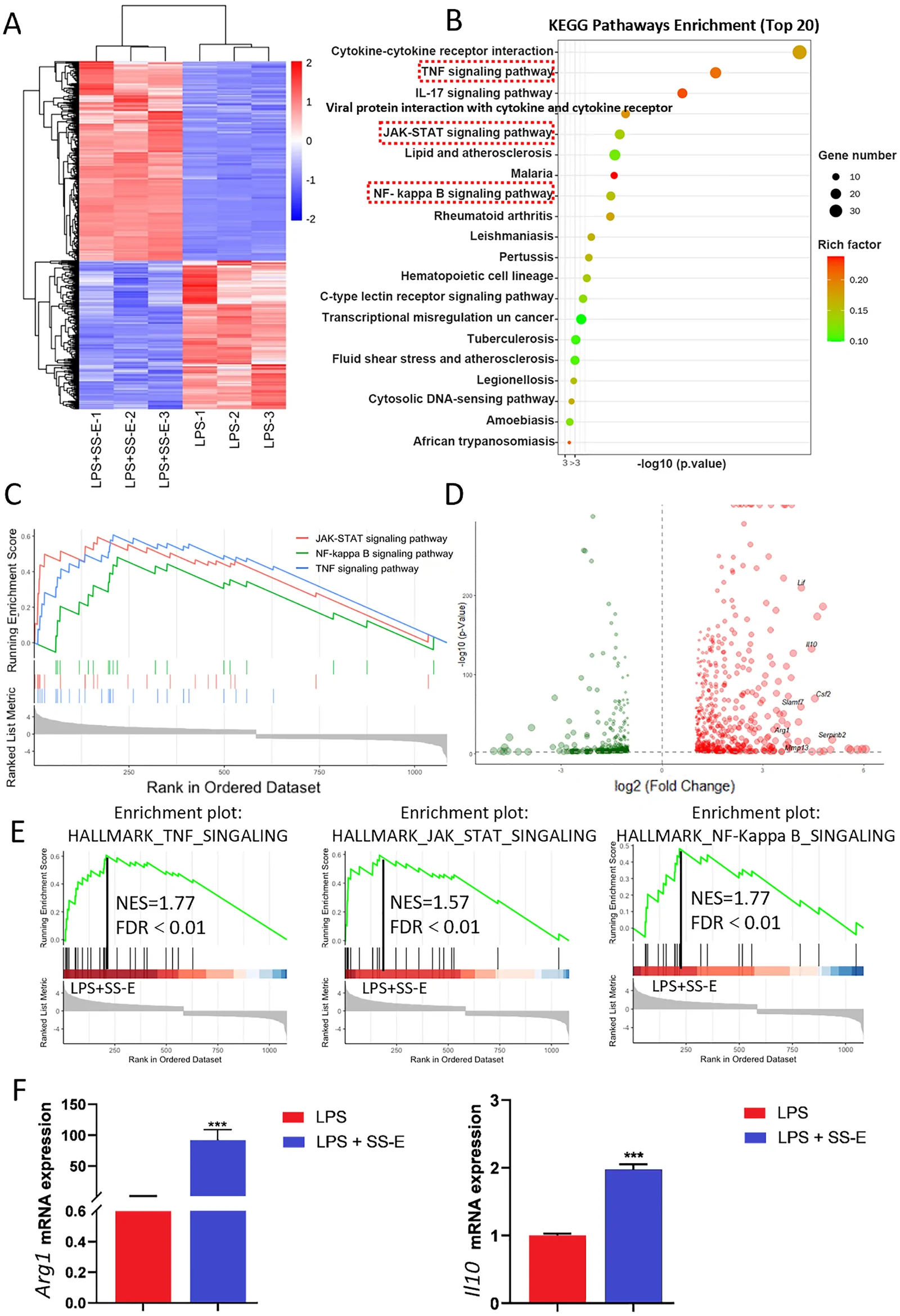
SS-E modulates the inflammatory gene expression profile of LPS-polarized macrophages. (A) Heatmap of differentially expressed genes in LPS-stimulated inflammatory macrophages. (B) KEGG pathway enrichment analysis of differentially expressed pathways. (C) The Gene Set Enrichment Analysis (GSEA) plot shows significant enrichment of gene sets in the LPS+SS-E-treated group (NES = 1.65, FDR q < 0.05). The peak of the green enrichment score plot on the left indicates activation of this pathway in the LPS+SS-E treated group. (D) Volcano plot of differentially expressed genes (DEGs). (E) GSEA revealed enrichment of inflammation-related signaling pathways, including TNF, JAK-STAT and NF-κB, in macrophages treated with SS-E following LPS polarization. (F) RT-qPCR analysis of *Arg1* and *Il-10* expression in LPS-stimulated inflammatory macrophages (n = 3). Significant differences between the groups, *P<0.05, **P<0.01, ***P<0.001.

Transcriptome sequencing of LPS-stimulated inflammatory macrophages revealed that SS-E treatment resulted in 661 upregulated and 520 downregulated genes compared to the LPS group (Figure 3A, D). Among the top upregulated genes, *Il10* and *Arg1* were most relevant to inflammatory regulation, along with several pro-inflammatory genes including *Il12b, Csf2*, and *Il6* (Table 2). Pathway analysis showed significant enrichment of classical inflammatory signaling pathways, including NF-κB, TNF, and JAK-STAT (Figure 3B, C), with GSEA highlighting the NF-κB and TNF pathways as most significantly enriched (Figure 3E). RT‒qPCR confirmed the upregulation of *Il10* and *Arg1* (Figure 3F), consistent with the shift toward an M2-like phenotype observed in our functional assays, alongside the concurrent expression of pro-inflammatory genes revealed by transcriptomic analysis.

**Table 2:** Top 24 differentially expressed genes information after SS-E treatment of LPS-stimulated inflammatory macrophages.

Gene ID	Gene symbol	Log2FC
ENSMUSG00000042212	*Sprr2d*	6.06
ENSMUSG00000051076	*Vtcn1*	5.96
ENSMUSG00000004296	*Il12b*	5.82
ENSMUSG00000004948	*Zp3*	5.60
ENSMUSG00000062345	*Serpinb2*	5.05
ENSMUSG00000091255	*Speer4e*	4.71
ENSMUSG00000038067	*Csf3*	4.61
ENSMUSG00000018916	*Csf2*	4.55
ENSMUSG00000008461	*Fut1*	4.46
ENSMUSG00000016529	*Il10*	4.45
ENSMUSG00000034394	*Lif*	4.14
ENSMUSG00000038179	*Slamf7*	4.13
ENSMUSG00000046318	*Ccbe1*	4.05
ENSMUSG00000051748	*Wfdc21*	4.04
ENSMUSG00000021367	*Edn1*	3.90
ENSMUSG00000054905	*Stfa3*	3.86
ENSMUSG00000037762	*Slc16a9*	3.81
ENSMUSG00000038540	*Tmc3*	3.78
ENSMUSG00000035513	*Ntng2*	3.76
ENSMUSG00000050578	*Mmp13*	3.76
ENSMUSG00000025746	*Il6*	3.73
ENSMUSG00000019987	*Arg1*	3.72
ENSMUSG00000025481	*Urah*	3.71
ENSMUSG00000116872	*Gm6815*	3.69

### SS-E-CM promotes osteogenic differentiation of BMSCs in vitro

**Fig. 4:**
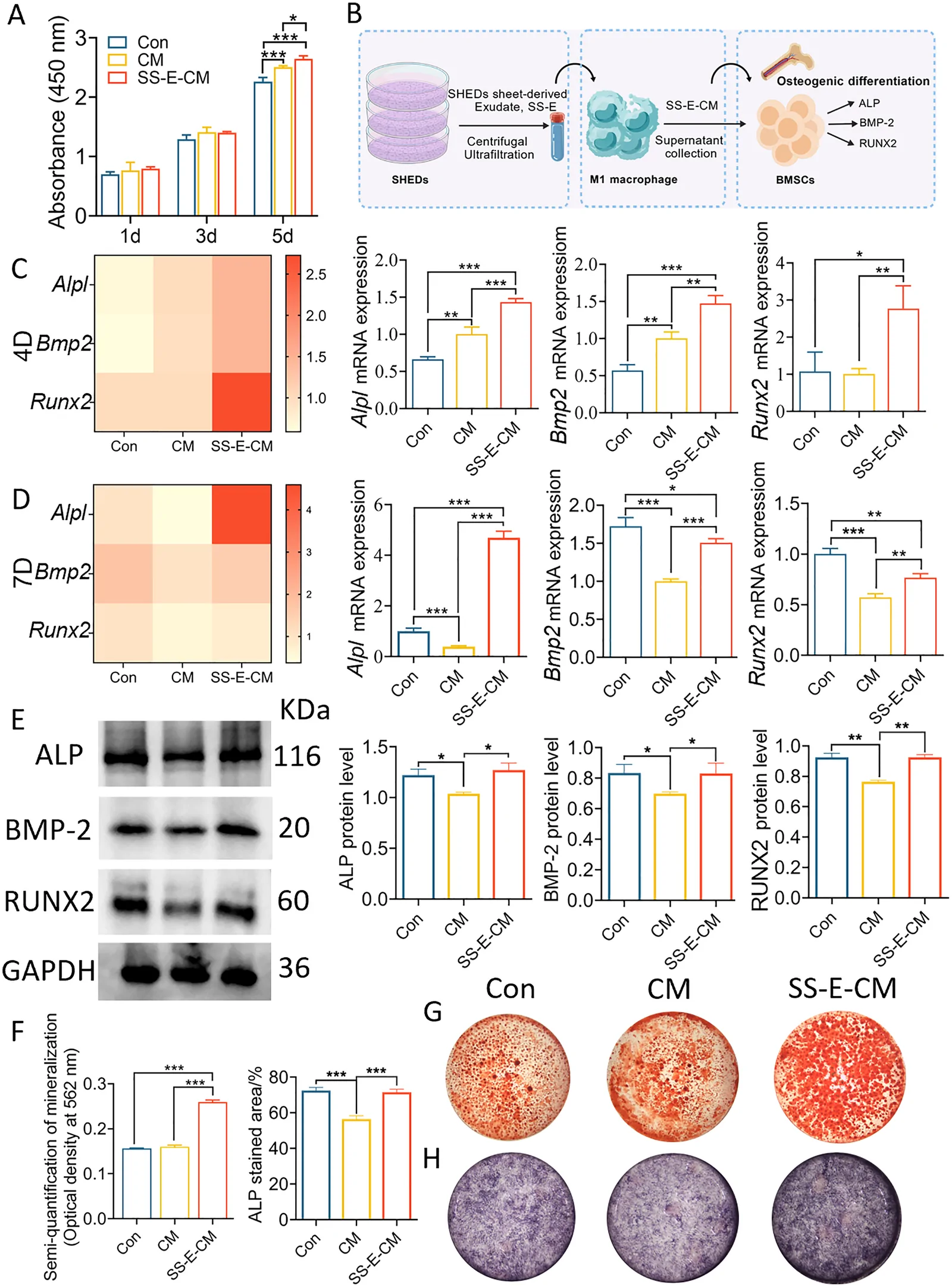
Effect of SS-E-CM on the viability and osteogenic differentiation of BMSC. (A) Cell viability was analyzed by CCK-8 assay (n = 3). (B) Schematic diagram for the collection of SS-E-CM. (C) RT-qPCR analysis of osteogenic differentiation in BMSC on day 4 (n = 3). (D) RT-qPCR analysis of osteogenic differentiation in BMSC on day 7 (n = 3). (E) Representative western blot images (left), and quantification (right) of osteogenic differentiation markers (n = 3). Representative images of alizarin red S staining (G) and ALP staining (H), and their quantitative analysis (F) (n = 3). Significant differences between the groups, *P<0.05, **P<0.01, ***P<0.001.

CCK-8 assays showed that SS-E-CM promoted the highest level of BMSC proliferation by day 5, outperforming both the Con and CM groups (Figure 4A). The process for collecting SS-E-CM is outlined in Figure 4B.

RT‒qPCR analysis on day 4 revealed that both CM and SS-E-CM upregulated the expression of *Alpl* and *Bmp2* compared to Con (Figure 4C). While *Runx2* was unchanged in the CM group, it was markedly elevated by SS-E-CM. By day 7, the expression of all osteogenic genes (*Alpl, Bmp2, Runx2*) decreased in the CM group but continued to rise in the SS-E-CM group (Figure 4D), demonstrating that SS-E-CM significantly promotes osteogenic differentiation.

Western blot analysis further supported these findings at the protein level on day 7 (Figure 4E). Both the Con and SS-E-CM groups showed higher protein expression of ALP, BMP-2, and RUNX2 compared to the CM group, with the most pronounced upregulation observed in the SS-E-CM group.

ARS staining results (Figure 4F, G) revealed that, after 14 days, the SS-E-CM group exhibited more numerous and darker-stained mineralized nodules compared to both the CM and Con groups, although no significant difference was observed between the Con and CM groups. Semi-quantitative CPC analysis further confirmed these results. ALP staining (Figure 4F, H) demonstrated that staining intensity was weaker in the CM group than in the Con group, while the SS-E-CM group showed the darkest staining among all groups. Collectively, these results suggest that macrophage inflammation modulated by SS-E promotes the osteogenic differentiation of BMSCs.

### SS-E promotes mandibular bone defect repair

**Fig. 5:**
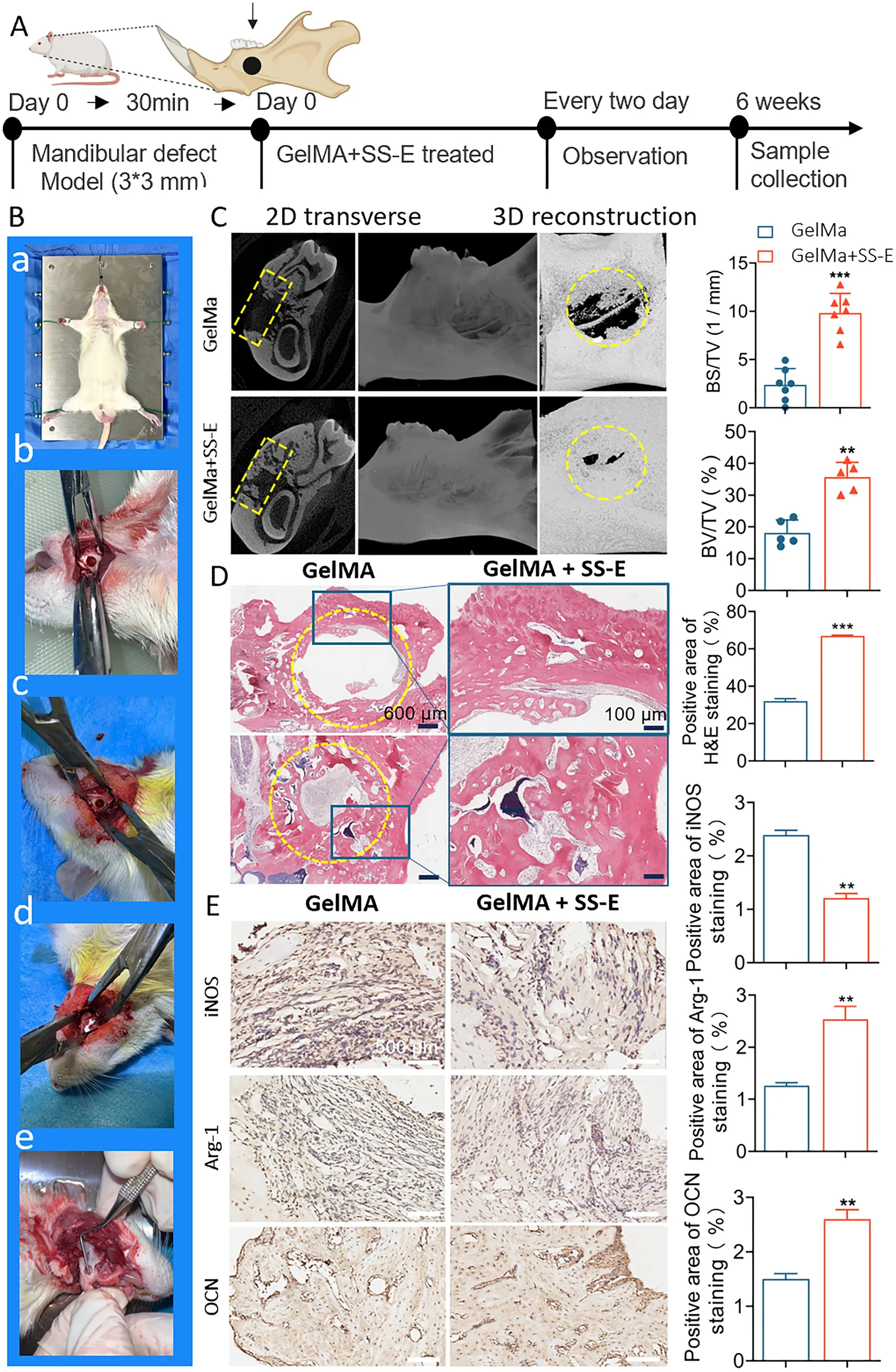
GelMA hydrogel loaded with SS-E promotes bone repair in rat mandibular defects. (A) Surgical procedure flowchart diagram. (B) (a) surgical preparation, (b) and (c) images of mandibular defect modeling from different angles, (d) material implantation, (e) periodontal probe indicating the defect size. (C) Representative micro-CT sectional view of the healing of mandibular bone defect in rats and quantitative analysis of newly formed bone parameters (n = 7). (D) Representative HE staining images (Left) and quantitative results (Right) (n = 7). (E) Representative microscopic images (Left) of immunohistochemistry of tissue sections and quantification (Right) of iNOS, Arg-1, and OCN expression (n = 7). Significant differences between the groups, **P<0.01, ***P<0.001.

SS-E loaded in GelMA was applied to rat mandibular defects (GelMA vs GelMA+SS-E; n = 7). The surgical procedure and timeline are summarized in Figure 5A, B.

Micro-CT analysis revealed enhanced bone healing in the GelMA+SS-E group compared to the GelMA-alone group, as visualized in both 2D and 3D reconstructions (Figure 5C, defect areas indicated by yellow dashed lines). Quantitative analysis of the 3D micro-CT data further demonstrated that the GelMA+SS-E group exhibited significantly increased bone volume fraction (BV/TV) and bone surface density (BS/TV) relative to the GelMA group (Figure 5C).

Histological evaluation with H&E staining (Figure 5D, left) indicated substantially enhanced new bone formation in the GelMA+SS-E group. While the control group exhibited large defect areas with limited repair and inflammatory cell infiltration, the GelMA+SS-E group showed continuous new bone formation along the defect margin, greater osteoid thickness, and abundant osteoblast alignment with evident bone matrix deposition. Most of the GelMA hydrogel had degraded by 6 weeks in both groups, with minimal residual material stained blue. Quantitative histomorphometric analysis confirmed significantly higher new bone volume in the GelMA+SS-E group (Figure 5D, right).

Immunohistochemical staining (Figure 5E) demonstrated markedly reduced expression of iNOS in the GelMA+SS-E group compared to the GelMA group. In contrast, anti-inflammatory Arg-1 and osteogenic marker OCN were upregulated. These results indicate that SS-E facilitates bone regeneration and attenuates macrophage-mediated inflammation in mandibular defects.

## Discussion

This study investigated the immunomodulatory capacity of SS-E, a concentrated secretome derived from the supernatant of osteogenically induced SHED sheets, in bone defect repair. Our findings demonstrate that SS-E effectively attenuates the LPS-induced pro-inflammatory response in macrophages, promoting a phenotypic shift toward an M2-like state characterized by upregulation of Arg-1, IL-10, and CD206, along with downregulation of iNOS. SS-E-CM enhanced the osteogenic differentiation of BMSC *in vitro*, and local delivery of SS-E via a GelMA hydrogel scaffold significantly improved bone regeneration in a rat mandibular defect model. Collectively, these results support our hypothesis that SS-E modulates the osteoimmune microenvironment to favor bone repair, establishing it as a promising cell-free strategy for craniofacial reconstruction.

Transcriptomic analysis of SS-E-treated macrophages revealed a gene expression profile far beyond the simplistic binary division between pro-inflammatory and pro-reparative macrophage states under LPS stimulation. While canonical anti-inflammatory markers such as *Il10* and *Arg1* were upregulated, consistent with reparative macrophage function, we also detected concurrent elevation of classic transcripts including *Il12b, Csf2*, and *Il6*. This co-upregulation of opposing gene sets is consistent with well-documented macrophage phenotypic plasticity and the formation of hybrid chimeric transcriptional profiles in tissue injury microenvironments, where macrophages rarely maintain a single fixed functional state. Transient mild expression of pro-inflammatory mediators acts as an essential trigger to initiate tissue repair, whereas prolonged excessive inflammation drives tissue damage. This mixed expression pattern suggests that SS-E induces a nuanced, transient activation state instead of locking macrophages into terminal polarization. Such dynamic plasticity is typical of macrophages exposed to complex secretome-derived stimuli,^15^ and the coexistence of pro- and anti-inflammatory signals facilitates ordered tissue repair: a brief controlled inflammatory burst followed by timely inflammation resolution is indispensable for robust regeneration.^16^ Elucidating the spatiotemporal transcriptional dynamics in vivo will clarify how SS-E remodels a favorable osteoimmune microenvironment. Consistent with these in vitro observations, our in vivo histological data verify that SS-E alleviates persistent local inflammation.

The effects of SS-E-CM on BMSC osteogenesis *in vitro*, while overall positive, exhibited temporal variability across different experimental endpoints. Notably, *Bmp2* mRNA and RUNX2 protein levels in the SS-E-CM group were lower than controls at certain time points, despite comparable or enhanced protein expression of other markers (Figure 4C, D, E). This apparent discrepancy likely reflects the temporally regulated nature of osteogenic differentiation, which proceeds through sequential waves of transcription factor activation and matrix protein deposition.^17^^,^^18^ It is plausible that SS-E-CM accelerates the differentiation kinetics, shifting the peak expression of early osteogenic genes to an earlier time point, thereby yielding lower relative expression when measured at a fixed, later endpoint. The modest but significant enhancement of matrix mineralization, as evidenced by Alizarin Red and ALP staining, together with the consistent improvement in *in vivo* bone formation, substantiates the pro-osteogenic capacity of SS-E-CM. Future time-course studies with higher sampling frequency will be necessary to fully characterize the dynamic effects of SS-E-CM on BMSC differentiation kinetics.

The term 'SS-E' was deliberately chosen to denote a concentrated preparation derived specifically from the supernatant of SS, distinguishing it from standard conditioned medium (CM), which is typically unconcentrated and obtained from conventionally cultured MSC. This distinction is conceptually significant, as three-dimensional sheet culture under osteogenic induction may enrich the secretome with a unique repertoire of regenerative and immunomodulatory factors relative to conventional monolayer cultures.^8^ To ensure quality control, we used BCA total protein quantification to standardize SS-E dosage. Although comprehensive proteomic profiling and EV characterization were not performed herein, accumulating evidence has confirmed that 3D cell sheet culture remodels secretory profiles and enriches trophic, immunomodulatory and osteogenic factors compared with conventional 2D culture. The distinct bioactivity of SS-E observed in our *in vitro* and *in vivo* assays indirectly supports the notion that osteogenically induced SHED sheets secrete unique functional mediators; comprehensive component identification via LC-MS/MS and EV characterization will form the core of our subsequent mechanistic investigations. Within the landscape of cell-free therapeutics, SS-E occupies a distinct niche. It offers greater complexity and potential synergistic effects compared to purified EV, as it retains the full complement of soluble proteins, growth factors, and vesicular components.^19^ Simultaneously, it circumvents the safety concerns associated with whole-cell therapies, including immune rejection and tumorigenicity, by virtue of its acellular nature.^5^ However, this compositional complexity also presents challenges for standardization, quality control, and mechanistic deconvolution. The use of GelMA hydrogel as a delivery vehicle in this study effectively addressed the issue of rapid *in vivo* clearance, enabling sustained local exposure to SS-E, a critical consideration for future translational applications.

This study adopted a xenogeneic experimental design, where human-derived SS-E was applied to murine RAW264.7 macrophages, rat BMSC, and rat mandibular bone defects. As a fully acellular secretome product, SS-E contains no intact cell membranes or xenogeneic cellular antigens, thereby conferring extremely low immunogenicity compared with live cell transplantation.^20^ Most core immunomodulatory and osteogenic functional factors within SS-E (BMP-2, TGF-β, IL-10) are highly conserved across mammalian species, and abundant preclinical data confirm that human stromal cell secretomes can bind rodent receptors and execute conserved biological functions.^21^ Consistent with our *in vivo* H&E and immunohistochemical readouts, no obvious xenogeneic rejection or excessive inflammatory response was detected within rat bone defects. We acknowledge the inherent limitations of rodent xenogeneic models, and further validation using homologous large-animal models will be conducted prior to clinical translation.

Several limitations should be acknowledged. First, mechanistic insights are primarily correlative; definitive causal evidence requires loss- and gain-of-function studies (eg, Arg-1 knockdown or inhibition) to establish necessity and sufficiency. Second, the specific bioactive components within SS-E responsible for its immunomodulatory effects remain unidentified; systematic fractionation coupled with proteomic and functional analyses is needed to deconvolute its composition and enable quality control. Third, while the rat mandibular defect model is a standard preclinical platform, translational validation in larger animal models (eg, swine or non-human primates) is necessary to account for species-specific differences in bone physiology and healing kinetics. Addressing these limitations will be critical for advancing SS-E toward clinical translation.

## Conclusion

In summary, this study demonstrates that SS-E promotes bone regeneration by modulating the osteoimmune microenvironment. SS-E attenuates the pro-inflammatory response in macrophages and facilitates their transition toward a pro-reparative phenotype, thereby supporting the osteogenic differentiation of BMSC. When delivered via a GelMA hydrogel scaffold in a rat mandibular defect model, SS-E effectively reduced local inflammation and enhanced new bone formation. These findings establish SS-E as a promising cell-free immunomodulatory strategy for scaffold-based craniofacial bone tissue engineering and provide a foundation for further mechanistic inquiry and translational development.

## Author contributions

X.X., W.W., and W.H. contributed equally as co-first authors and conducted most experiments and data analysis. D.X., J.Z., K.L., Y.R., L.W., and C.H. provided technical and experimental support. J.L.P., S.Z., and B.P. supervised the project as co-corresponding authors. All authors reviewed and approved the manuscript.

## Ethics approval and consent to participate

Animal experiments complied with ARRIVE 2.0 guidelines and received ethical approval (No. 20210903) from the Ethics Committee of Guangdong Huawei Testing Co, Ltd. SHEDs were isolated from 6 to 8-year-old children after obtaining parental written informed consent and children’s verbal assent. The human sample collection was approved by the Ethics Committee of the Affiliated Stomatological Hospital of Guangzhou Medical University (No. JCYJ2022027).

## Funding

This work was supported by the Guangdong Medical Research Fund (A2024556, A2024158); Guangzhou Medical University Research Ability Enhancement Project (2025SRP027); Key Laboratory of Guangdong Higher Education Institutes (2021KSYS009); and the Higher Education Scientific Research Project for Graduate Students of the Guangzhou Municipal Education Bureau (2024312073).

## Availability of data and materials

The sequencing datasets generated during the current study are deposited in the NCBI Sequence Read Archive (SRA) under BioProject ID PRJNA1357866 (SRA Study ID: SRP643125, Submission ID: SUB15752419), accessible at https://www.ncbi.nlm.nih.gov/bioproject/PRJNA1357866.

## Conflict of interest

None disclosed.

## Appendix Supplementary materials

Image, application 1

Image, application 2

## Footnotes

Supplementary material associated with this article can be found in the online version at doi:10.1016/j.identj.2026.109793.
