# Supplementary material for: Secretome of SHED Sheets Facilitates Mandibular Bone Defect Repair by Shifting Macrophages Inflammatory State
Source: Int Dent J. 2026 Aug 18;76(5):109793. doi: 10.1016/j.identj.2026.109793 (PMC13519614; doi:10.1016/j.identj.2026.109793)
Supplement: Supplementary file 1 [file mmc1.pdf]

Supplementary Table 1. MIQE checklist			
**Experimental design**		**qPCR oligonucleotides**	
Definition of experimental and control groups	Grouping was performed according to different cell types; details are provided in the main text	Primer sequences	Listed in Table 1 of the manuscript
Number within each group	At least 3 biological replicates per group	RTPrimerDB identification number	N/A
Assay carried out by the core or investigator's laboratory?	Guangzhou Key Laboratory of Basic and Applied Research of Oral Regenerative Medicine, Guangzhou Medical University, Guangzhou, China.	Probe sequences	N/A - SYBR Green I assay
Acknowledgment of authors' contributions	See the author contributions section	Location and identity of any modifications	No modifications
**Sample**		Manufacturer of oligonucleotides	Guangzhou Generay Biotech Co., Ltd., China
Description	RAW264.7 cells or BMSCs	Purification method	PAGE purification
Volume/mass of sample processed	1×10^6 cells per sample	**qPCR protocol**	
Microdissection or macrodissection	N/A - cultured cells	Complete reaction conditions	SYBR Premix Ex Taq™ (Tsingke, China, Cat#TSE201), 20 µL reaction
Processing procedure	Cells were lysed according to the manufacturer's protocol of the SteadyPure Quick RNA Extraction Kit (Accurate Biotechnology, China),	Reaction volume and amount of cDNA/DNA	20 µL, 2 µL cDNA template (1:5 dilution)

	and RNA extraction was immediately performed		
If frozen, how and how quickly?	Cell lysates were processed immediately without freezing	Primer, (probe), Mg^{2+}, and dNTP concentrations	Primers 0.4 µM each; Mg^{2+} and dNTP included in master mix
If fixed, with what and how quickly?	N/A - fresh lysates	Polymerase identity and concentration	Taq DNA Polymerase, included in SYBR Premix
Sample storage conditions and duration (especially for FFPE[b] samples)	N/A - processed immediately	Buffer/kit identity and manufacturer	SYBR Premix Ex Taq™, Tsingke, China
**Nucleic acid extraction**		Exact chemical composition of the buffer	As per the manufacturer's formulation
Procedure and/or instrumentation	SteadyPure Quick RNA Extraction Kit (Accurate Biotechnology, China, Cat#AG21022)	Additives (SYBR Green I, DMSO, and so forth)	SYBR Green I included in the master mix
Name of kit and details of any modifications	SteadyPure Quick RNA Extraction Kit, used according to the manufacturer's protocol without modification	Manufacturer of plates/tubes and catalog number	96-well plate, Bio-Rad, Cat#HSP9601
Source of additional reagents used	None	Complete thermocycling parameters	95°C 30 sec; (95°C 5 sec, 60°C 30 sec, 72°C 30 sec) × 40 cycles; melt curve 65-95°C
Details of DNase or RNase treatment	On-column DNase I digestion is included in the kit	Reaction setup (manual/robotic)	Manual pipetting
Contamination assessment (DNA or RNA)	Assessed by no-reverse transcription (no-RT) control	Manufacturer of qPCR instrument	Bio-Rad CFX96 Touch Real-Time PCR Detection System
Nucleic acid quantification		**qPCR validation**	

Instrument and method	NanoDrop 2000 spectrophotometer	Evidence of optimization (from gradients)	Annealing temperature gradient 58-62°C; optimal 60°C
Purity (A_{260}/A_{280})	1.8-2.0	Specificity (gel, sequence, melt, or digest)	Melt curve analysis: single peak confirmed
Yield	Mean 200-500 ng/µL	For SYBR Green I, Cq of the NTC	NTC Cq > 40 or no amplification
RNA integrity: method/instrument	1% agarose gel electrophoresis	Calibration curves with slope and y-intercept	Slope: -3.2 to -3.5; y-intercept: 30-35
RIN/RQI or C_q of 3' and 5' transcripts	28S/18S rRNA ratio > 1.5	PCR efficiency calculated from slope	90-110%
Inhibition testing (C_q dilutions, spike, or other)	Tested by a 1:5 dilution series	r^2 of calibration curve	> 0.99
**Reverse transcription**		Linear dynamic range	10^2 - 10^7 copies
Complete reaction conditions	PrimeScript® RT Reagent Kit (Takara, Japan, Cat#RR047A), 20 µL reaction	Cq variation at LOD	CV < 5%
Amount of RNA and reaction volume	1 µg total RNA, 20 µL reaction volume	CIs throughout range	95% CI
Priming oligonucleotide (if using GSP) and concentration	Random 6 mers (50 µM) and oligo dT primer (50 µM)	Evidence for LOD	10 copies/reaction
Reverse transcriptase and concentration	PrimeScript RTase, included in kit	If multiplex, efficiency and LOD of each assay	N/A - singleplex assays
Temperature and time	37°C for 15 min, 85°C for 5 sec	**Data analysis**	
Manufacturer of reagents and catalogue numbers	PrimeScript® RT Reagent Kit, Takara, Cat#RR047A	qPCR analysis program (source, version)	Bio-Rad CFX Manager 3.1
Cqs with and without reverse transcription	No-RT control: Cq > 40 or no amplification	Method of Cq determination	Automatic threshold method

Storage conditions of cDNA	-20°C, used within 1 month	Outlier identification and disposition	Replicates with SD > 0.5 excluded
**qPCR target information**		Results for NTCs	No amplification in all NTCs
Gene symbol	Mu-GAPDH, Mu-iNOS, Mu-Arg-1, Mu-IL-10, Ra-Runx-2, Ra-ALP, Ra-BMP-2, Ra-GAPDH , Mu- GAPDH	Justification of the number and choice of reference genes	Mu-GAPDH for mouse samples; Ra-GAPDH for rat samples; validated by literature as stable reference genes for RAW264.7 cells and BMSCs
Sequence accession number	NM_001289726.1; NM_001313922.1; NM_007482.3; NM_010548.2; NM_001278484.3; NM_013059.3; NM_017178.2; NM_017008.4; NM_001289726.1	Description of normalization method	$2^{-\Delta\Delta Cq}$ method
Location of amplicon	N/A - primers designed to span exons where possible	Number and concordance of biological replicates	3 biological replicates per group
Amplicon length	111 bp (*Mu-GAPDH*); 105 bp (*Mu-Inos*); 147 bp (*Mu-Arg1*); 119 bp (*Mu-Il10*); 182 bp (*Ra-Runx2*); 81 bp (*Ra-Alpl*); 99 bp (*Ra-BMP2*); 206 bp (*Ra-GAPDH*)	Number and stage (reverse transcription or qPCR) of technical replicates	Triplicate at the qPCR stage
In silico specificity screen (BLAST, and so on)	Verified by NCBI BLAST	Repeatability (intra-assay variation)	Intra-assay CV < 3%

Pseudogenes, retropseudogenes, or other homologs?	No known pseudogenes for target genes	Reproducibility (interassay variation, CV)	Inter-assay CV < 5%
Sequence alignment	Aligned using NCBI Primer-BLAST	Power analysis	N/A - exploratory study
Secondary structure analysis of amplicon	Analyzed by NCBI Primer-BLAST	Statistical methods for the significance of results	Comparisons between two groups were assessed by Student's t-test, while one-way ANOVA was used for comparisons among three or more groups, GraphPad Prism 9.5.1
Location of each primer by exon or intron (if applicable)	Primers designed to span exon-exon junctions where possible	Software (source, version)	GraphPad Prism 9.5.1
What splice variants are targeted?	All known splice variants	CQ or raw data submission with RDML	Available upon request
