# Supplementary material for: Secretome of SHED Sheets Facilitates Mandibular Bone Defect Repair by Shifting Macrophages Inflammatory State
Source: Int Dent J. 2026 Aug 18;76(5):109793. doi: 10.1016/j.identj.2026.109793 (PMC13519614; doi:10.1016/j.identj.2026.109793)
Supplement: Supplementary file 2 [file mmc2.pdf]

The ARRIVE Essential 10		
**Study design**	1	**a.** Experimental groups: GelMA and GelMA + SS-E<br>**b.** The experimental unit was the individual rat.
**Sample size**	2	**a.** This study allocated 7 rats per group, using a total of 14 rats.<br>**b.** The sample size was determined based on previously published studies with similar research objectives and designs. To ensure adequate statistical power in this experiment, the sample size was set to 7 rats per group.
**Inclusion and exclusion criteria**	3	**a.** The inclusion and exclusion criteria for this study were pre-established. The inclusion criterion was: rats in good health throughout the experiment that successfully completed all modeling and intervention procedures.<br>The exclusion criteria included: individuals that died during the experiment due to non-experimental factors (e.g., anesthesia accident, modeling accident, infection); individuals for whom severe technical failure during intervention or detection led to unobtainable or clearly invalid data; individuals identified via blinded analysis as having extreme outliers in the measurement of a certain key indicator (using Grubbs' test, $p < 0.05$).<br>**b.** No animals were excluded in this experiment, as all animals remained healthy until the endpoint and did not meet the exclusion criteria.<br>**c.** A total of 14 rats were purchased, all 14 underwent modeling, and all 14 were euthanized at the endpoint.
**Randomisation**	4	**a.** Group allocation was performed using a computer-generated random number sequence. All animals were numbered sequentially after acclimatization. Random numbers corresponding to the animal numbers were generated using SPSS statistical software (version 25.0). These random numbers were sorted in ascending order, with the first half assigned to the control group and the second half to the experimental group.<br>**b.** To minimize potential confounding factors, the following strategies were implemented:<br>Allocation concealment: The randomization sequence was generated and maintained by a researcher not involved in animal husbandry or intervention. The researcher performing the grouping only became aware of the group assignments immediately before the intervention.<br>Environmental control: All animals were housed in the same room under uniform temperature, humidity, and light/dark cycle conditions. Cage positions were systematically rotated

		weekly to eliminate effects of environmental location differences.<br>Blinding: Personnel conducting subsequent experimental interventions (e.g., drug administration) and outcome assessment (e.g., histological analysis) were blinded to the group allocations.
**Blinding**	5	Awareness of group information at different experimental stages was as follows:<br>Allocation and reagent preparation stage: The experimental designer [B.P.] generated the randomization sequence and prepared the GelMA hydrogel into two formulations accordingly: 'GelMA-A' (containing PBS, control) and 'GelMA-B' (containing SS-E, experimental) . After delivering the labeled formulations to the surgeon, this designer took no part in subsequent procedures.<br>Experimental implementation stage: The surgeon performing the mandibular bone defect modeling and implantation of the GelMA hydrogel/biofilm knew only whether they were implanting 'GelMA-A' or 'GelMA-B' but was completely unaware of which code corresponded to which group. All surgical instruments and procedures were identical between the two groups.<br>Outcome assessment stage: Blinding was strictly implemented for the assessment of all outcomes. All researchers responsible for the following evaluations were blinded to group information: imaging evaluation (e.g., Micro-CT scanning and 3D reconstruction analysis); histological section staining (e.g., H&E staining) and scoring; quantitative analysis of immunohistochemistry/immunofluorescence staining.<br>Data analysis stage: The researcher performing the statistical analysis used the coded data (i.e., Group A vs. Group B). The blinding code was only broken by the experimental designer after the final statistical results were obtained, decoding Groups A/B into the experimental and control groups.

**Outcome measures**	6	**a.** The outcome measures assessed in this study included:<br>Radiographic indicators: Evaluated by micro-computed tomography (Micro-CT), including bone volume fraction (BV/TV), Bone Surface / Total Volume (BS/TV).<br>Histomorphometric indicators: Evaluated by Hematoxylin and Eosin (H&E) staining to assess new bone formation area, collagen fiber arrangement, and maturity.<br>Histochemical indicators: Evaluated by immunohistochemistry (IHC) staining to assess the expression levels and localization of inflammation and osteogenesis-related markers (such as Arg-1 and OCN).<br>**b.** The primary outcome measure was the bone volume fraction (BV/TV) analyzed by Micro-CT, which is the gold standard for assessing bone regeneration efficacy. The sample size for this study was determined based on a priori calculations designed to detect intergroup differences in this indicator.
**Statistical methods**	7	**a.** All experiments were performed in triplicate, and data are presented as mean ± standard deviation (SD). Analyses were conducted using GraphPad Prism 9.5.1 (GraphPad Software, USA). Comparisons between two groups were made using the Student's t-test, while one-way ANOVA was applied for comparisons among three or more groups. A significance threshold of $\alpha=0.05$ was used, and differences with $p < 0.05$ were considered statistically significant.<br>**b.** Before conducting parametric tests (such as t-test, ANOVA), we tested the data for normality and homogeneity of variances.<br>Normality test: Using the Shapiro-Wilk test.<br>Homogeneity of variances test: Using Levene's test.<br>Method modification: If the data did not meet the normality assumption, the Mann-Whitney U test was used for comparisons between two groups; if the data did not meet the assumption of homogeneity of variances, Welch's corrected t-test or non-parametric tests were applied.

**Experimental animals**	8	Details of the experimental animals used in this study are as follows:<br>Species, strain, and substrain: Sprague-Dawley rats, abbreviated as SD rats.<br>Sex and number: Male, totaling 14 rats.<br>Developmental stage, age, and weight: Upon arrival at the laboratory, the animals were in the juvenile stage at 42 to 49 days old. Before surgery, their body weights were confirmed to be in the range of 210-240g, with no statistically significant difference between the two groups.<br>Microbiological status: SPF level, meaning specific pathogen-free.<br>Source: All animals were purchased from Southern Medical University (Production License No.: SCXK (Yue) 2021-0041), with a quality certificate issued by the Guangdong Laboratory Animals Monitoring Institute (No.: 44002100032204), ensuring clear genetic background and health status.<br>Health status and genetic background: Based on their SPF status and the quality certificate provided by the supplier, all animals were in good health at the start of the experiment and free of specific pathogens. SD rats are an outbred stock with a stable genetic background and are commonly used in orthopaedic research.<br>Housing and husbandry: Animals were acclimatized for at least 7 days in the animal facility of the experimental unit (Guangdong Huawei Testing Co., Ltd.) (Use License No.: SYXK (Yue) 2021-0913). The housing environment was maintained under standard conditions (temperature 22 ± 2°C, relative humidity 50 ± 10%, 12/12-hour light/dark cycle), with free access to food and water.<br>Previous use: This was the first experimental use of the animals.
**Experimental procedures**	9	**a.** SPF-grade SD rats were selected. A critical-sized bone defect measuring 3×3×3 mm was created in the unilateral mandibular molar region. The experimental group received GelMA hydrogel containing SS-E at a concentration of 1.6 mg/mL, while the control group received GelMA hydrogel containing an equal volume of PBS. The hydrogel was implanted into the defect and covered with a bio-membrane. Subsequently, local drug administration was performed twice weekly for 2 weeks: the experimental group received injections of 200 μL of SS-E (1.6 mg/mL), and the control group received an equal volume of sterile PBS. At 6 weeks post-operation, the rats were euthanized, and the complete mandibles were collected for Micro-CT scanning. Following decalcification, the bone tissues

		underwent H&E staining analysis and immunohistochemical analysis to assess inflammation and osteogenic indicators. **b.** The surgery was performed once. Local drug injections were then administered twice per week for 2 consecutive weeks. Rats were euthanated via CO_2 at 6 weeks post-operation. **c.** The laboratory experiments were applied for and conducted at the Guangdong Huawei Testing Co., Ltd.. **d.** The experimental design was based on the following rationale:<br>Animals and Model: SD rats are a common model for bone defect repair research, and their mandibular physiology shares certain similarities with humans. The 3×3×3 mm defect is widely validated as a critical-sized bone defect that is difficult to heal spontaneously, thus enabling effective evaluation of the osteogenic potential of implanted materials or drugs.<br>Materials and Grouping: GelMA hydrogel was chosen due to its good biocompatibility, injectability, and three-dimensional network structure, allowing it to serve as a drug carrier and simulate the extracellular matrix. The PBS-containing GelMA group was established as the control to exclude the effect of the hydrogel itself on bone regeneration, thereby independently evaluating the therapeutic effect of the SS-E.<br>Dosing Regimen: Local drug injections were continued after hydrogel implantation to maintain sufficient drug concentration in the defect area and continuously stimulate the bone regeneration process. The dosage and frequency were based on prior in vitro cell experiments or pilot study results indicating its efficacy and safety.<br>Endpoint and Evaluation Methods: The 6-week post-operation time point was chosen as the endpoint based on literature reports and pilot experiments, indicating this duration is sufficient to observe early to mid-term repair processes in rat mandibular defects. Micro-CT was used for quantitative analysis of the three-dimensional structure of new bone formation; H&E staining was used to observe general tissue morphology and nascent bone tissue; immunohistochemistry was used for the localization and semi-quantification of key osteogenesis-related markers at the protein level, thereby confirming the treatment effect from morphology to molecular mechanisms across multiple levels.
**Results**	10	For each quantitative outcome measure in each experimental group, we reported the sample size (n).
